# Racial/ethnic differences in the relationship between wealth and health across young adulthood

**DOI:** 10.1016/j.ssmph.2022.101313

**Published:** 2022-12-20

**Authors:** Sicong Sun, Hedwig Lee, Darrell L. Hudson

**Affiliations:** a School of Social Welfare, The University of Kansas, 1545 Lilac Lane, Lawrence, KS, 66045, USA; b Department of Sociology, Trinity College of Arts & Sciences, 417 Chapel Dr, Durham, NC, 27708, USA; c Brown School, Washington University in St. Louis, Campus Box 1196, One Brookings Drive, St. Louis, MO, 63130, USA

**Keywords:** Race, Wealth, Racial health disparities, Social determinants of health, Young adults, Socioeconomic status

## Abstract

• Wealth attenuated racial differences in self-rated health during young adulthood.
• Wealth had consistent incremental effect on health among White & Hispanic Americans.
• For Black Americans, wealth was protective of health in the highest wealth quartile.
• Individual wealth, not parental wealth was associated with health among Hispanics.

## 1 Introduction

Wealth inequities across race/ethnicity are one of the most pressing social and economic issues in the United States. Due to sociohistorical factors, such as redlining and residential steering practices, there has been inequitable distribution of opportunities for economic security and mobility along race/ethnicity in the U.S. (Williams & Collins, 1995, 2001; Williams, Lawrence, & Davis, 2019). Wealth helps to protect people from economic shocks and ease critical transitions across the life course. Black Americans on average receive less intergenerational financial inheritance compared to White Americans and are far more likely to be downwardly mobile in household wealth (Oliver & Shapiro, 2006; Pfeffer & Killewald, 2019). Compared to the examination of other socioeconomic position (SEP) indicators, such as income and education—as their association with health and contributions to racial/ethnic health inequities have been thoroughly documented in the existing literature (Krieger et al., 1993; Link & Phelan, 1995; Williams & Collins, 1995)—wealth is an understudied SEP indicator in health.

Although the relationship between wealth and health has been less frequently studied compared to the relationship between income and health, there is a growing body of evidence that has revealed that greater levels of wealth predict better health outcomes. For example, in a systematic review of wealth–health associations Pollack et al. (2007) found that greater wealth was associated with better health, even after adjustments of other SEP indicators, and wealth explained portions of racial/ethnic disparities in health. Researchers have documented that indices of wealth are associated with a wide variety of health-related outcomes, including morbidity, mortality, functional status, mental health, self-rated health, and health related quality of life (Boen, 2016; Boen et al., 2020; Boen & Yang, 2016; Hajat et al., 2010, 2011).

Among the emerging research that has examined wealth and health longitudinally, most studies have examined middle age and later life samples (e.g., Boen et al., 2016; Boen & Yang; 2016; Brown, 2016). By the time a person transitions from young to middle adulthood, both social (e.g., formation of relationship and family) and SEP (e.g., whether entering college and/or labor market) paths begin to diverge and health behaviors (e.g., diet, physical activity, and engagement with health care) have crystalized (Widome et al., 2013). Despite the importance of wealth in relation to transitioning to adulthood (e.g., attending college, purchasing a home, starting a family) there is little known about the relationship between wealth and health during young adulthood. Findings from previous studies highlight that the effects of SEP on health begin to emerge much earlier in life, especially in young adulthood (Mossakowski, 2008). It is imperative to understand the longitudinal time-varying relationship between wealth and health across young adulthood within racial/ethnic groups to better inform social policy to build assets and address health equity in this critical life stage.

Among the existing research that has examined wealth-health associations, findings generally indicate that there is a positive wealth–health associations, even after adjusting for other SEP indicators such as income and education (Boen & Yang, 2016; Hajat et al., 2010, 2011; Pollack et al., 2007, 2013). In a systematic review on health studies that included wealth, Pollack and colleagues wrote that studies that examined wealth-health associations within racial/ethnic groups have focuses on Black and White or White and non-White populations (Pollack et al., 2007). Moreover, among studies that investigated racial/ethnic heterogeneities, results have been mixed. For example, Ostrove et al. (1999) documented that wealth, in addition to more traditional indicators of SES (education and household income), made a unique and significant contribution to explaining both physical and mental health. In addition, the relationship between wealth and health outcomes did not differ for African Americans and White Americans. In contrast, Rodriguez et al. (1999) found that wealth was a predictor of depression for White Americans but not for African Americans. Using longitudinal data from the Panel Study of Income Dynamics (PSID), Shuey and colleagues found that effects of wealth on health did not significantly differ and remain similar for Blacks and Whites over the life course (Shuey & Willson, 2008). Another study used the PSID data and found that less wealthy whites had higher risk of poor/fair health relative to their wealthy counterparts but no association between wealth and health among the nonwhite sample (Hajat et al., 2011).

Pollack et al. (2013) used data drawn from two cross-sectional surveys (the Survey of Consumer Finance and the Health and Retirement Study) to examine the association between wealth and self-rated health among each racial/ethnic group. They found that net worth was significantly associated with poor/fair health status within each racial/ethnic group except for the younger Hispanic population. They found that accounting for the effects of net worth attenuated the association between income and poor/fair health (except among older Hispanics). In a study lead by Hershman et al. (2015), using data drawn from insurance claims, the authors found that household wealth partly explained racial disparities in hormonal therapy adherence and discontinuation among women with early-stage breast cancer. Moreover, they uncovered a significant interaction between race and net worth, such that lower adherence was observed among Black women in the low net worth group but not in medium and high net worth groups. Sharma (2019) studied the wealth–health association among a sample of 906 older Black women using data from the Health and Retirement Study from 2008 to 2010. Sharma found no statistically significant associations between wealth and self-rated health among this sample. Sharma suggested that wealth may play a limited role in determining health for older Black women.

### 1.1 The present study

To address the current gaps of lacking longitudinal evidence and examination of racial/ethnic differences in the wealth–health associations, especially among young adults and Hispanic Americans, this study aims to (a) investigate how each time-varying SEP indicator (i.e., income, education, and employment) is associated with health across young adulthood; (b) assess the association between wealth and health, adjusting for other common SEP indicators; (c) determine whether there are racial/ethnic differences in the wealth–health association across the three largest racial/ethnic groups in the United States—non-Hispanic Black, non-Hispanic White, and Hispanic Americans.

## 2 Methods

### 2.1 Data and sample

Data for this study were drawn from National Longitudinal Survey of Youth (NLSY) 1997–2017, which consists of approximately 9,000 youth who were 12 to 16 years old in 1997. Hispanic and non-Hispanic Black Americans were oversampled. Because wealth is measured every 5 years in the survey, three data points were included—when respondents were aged 20, 25, and 30. The total sample consists of 8,984 individuals. The NLSY97 survey is sponsored and directed by the U.S. Bureau of Labor Statistics and managed by the Center for Human Resource Research at The Ohio State University. Interviews are conducted by the National Opinion Research Center at the University of Chicago (Bureau of Labor Statistics, U.S. Department of Labor, 2019).

The NLSY 1997 provides opportunity to investigate three innovations in racial/ethnic differences in the wealth–health associations: (a) this dataset provides a large representative sample of Hispanics, who are understudied in the existing literature on the relationship between wealth and health; (b) this dataset provides previously unavailable information on the longitudinal time-varying relationship between wealth and health across young adulthood—when respondents were aged 20, 25 and 30; and (c) We were able to distinguish parental net worth and young adults net worth in this dataset.

### 2.2 Measurements

#### 2.2.1 Race/ethnicity

Race/ethnicity was based on respondents’ self-identified racial/ethnic group at the baseline, categorized into four groups—non-Hispanic Black, non-Hispanic White, Hispanic, and other or multiracial non-Hispanic. We included these four racial/ethnic groups in the whole sample analysis. Because American Indian and Alaska Native, Asian or Pacific Islander, mixed race groups have smaller sample sizes, we could not perform stratified analyses in these samples. Three racial/ethnic groups are examined in the subgroup analyses: non-Hispanic White (*n* = 4,406), non-Hispanic Black (*n* = 2,335), and Hispanic (*n* = 1,901).

#### 2.2.2 Health

The primary dependent variable was self-rated health (SRH), measured on a 5-point Likert scale. In alignment with previous studies, we dichotomized SRH as fair or poor health vs. excellent/very good/good health, with fair/poor health status being the reference category (Pollack et al., 2013).

#### 2.2.3 Wealth

Two indices of wealth were calculated: respondents’ reported net worth and parent-reported parental net worth in 1997. Parental net worth was measured at the baseline in 1997. Parental net worth was calculated based on parent-reported all assets, (including home value, checking and savings, stocks and bonds, automobiles, retirement accounts, college savings accounts) minus all debts. Individual net worth measures the respondent’s total net worth when respondents turned 20, 25, and 30 years old. These are separate measures of respondents’ own wealth from parental wealth after the respondent meets the criteria of independency. NLSY97 youth were considered independent if they have had a child, were enrolled in a 4-year college, were no longer enrolled in school, were not living with any parents or parent-figures or had ever been married or were in a marriage-like relationship. Net worth was calculated as total assets minus total debt. Total assets include property, vehicles, businesses, pensions, and other types of financial (e.g., money in savings, CDs, stocks, and trusts) and non-financial assets. Total debt or liabilities include outstanding balances on credit cards, mortgages, lines of credit, vehicle debt, education debt, and other types of loans. Inflation was adjusted using the Consumer Price Index. All wealth was calculated in 2015 US dollars. We created wealth quartiles according to each racial/ethnic group for self-reported net worth when respondents were aged 20, 25, and 30, as well as the net worth of their family in 1997. For analyses using the entire sample, wealth quartiles were created according to wealth distribution of the whole sample. For racial/ethnic stratified analysis, however, a small number of Black and Hispanic adults would have been included in the highest wealth quartile if we created quartiles according to wealth distribution of the whole sample, which would have resulted in unstable estimates of the coefficients as well as limiting the implications. Following Pollack et al.’s (2013) approach, racial/ethnic group-specific cut-points were chosen to examine the association of wealth and health within racial ethnic groups in stratified analysis.

#### 2.2.4 Other SEP indicators

The other SEP indicators included household income (inflation adjusted in 2015 US dollars); education (a) high school and below, (b) associate/junior/bachelor’s degree, and (c) graduate/professional degree; and employment status (yes/no) at age 20, 25, and 30. These were included in the model as covariates. Using time-varying covariates enabled the study to capture the dynamic relationship between SEP indicators and health. Further, parental education (years of education of the highest educated parent) measured in 1997 was also controlled for as a time-invariant covariate.

#### 2.2.5 Sociodemographic controls

Sociodemographic factors that were adjusted for were gender (female/male), Census region (northeast, north central, south, west), geographic area (urban/rural), household size (number of people in the household) and health insurance (whether the respondent is insured or not at time of interview).

### 2.3 Analytic methods

All statistical analyses were performed in Stata 15 (StataCorp, 2017). The primary analytic strategy was Hierarchical Generalized Linear Model (Snijders & Bosker, 2012) because the data had a hierarchical nature where observations were collected multiple times. Two-level logistic regressions with random intercepts and time random slopes were performed to control for clustering effects where observations were nested within each individual. Multiple imputation by chained equations were used to impute missing predictor variables from the NLSY97 (Allison, 2001). We assumed the data is “missing at random,” rather than “missing completely at random” (Allison, 2001; Sterne et al., 2009). All variables, including dependent variables, in the analytic models were used for imputation. However, imputed values of the outcome variables were not used (Von Hippel, 2007). Twenty imputations per observation were produced, with relative efficiency above 99%. Missing data on the dependent variable were addressed with mixed-effect models, which used direct maximum likelihood estimation. Results were combined using Rubin’s rules (Grund, et al., 2016; Rubin, 1987).

To test whether wealth significantly added to the model given other common SEP indicators (e.g., education, income, employment) are already in the model, we ran two models using the whole sample. Model 1 included household demographic covariates as well as SEP indicators, namely income, education, employment, and parental education without net worth. Model 2 added respondents’ net worth quartiles and parental net worth in 1997, in addition to all SEP indicators and sociodemographic covariates in Model 1. In addition, this approach helped to determine whether the inclusion of wealth changed the association between education/income/employment and health. To test racial/ethnic differences in the relationship between wealth and health, we ran three subgroup analyses using non-Hispanic White, non-Hispanic Black, and Hispanic samples. Consistent with the whole sample analyses, our primary independent of variables of interest in stratified analyses were respondents’ net worth and parental net worth, with the same set of SEP indicators and sociodemographic indicators being adjusted for. All analyses applied NLSY weights to make results generalizable to the general population.

## 3 Results

### 3.1 Descriptive statistics of sample characteristics

Table 1 presents demographics of the respondents at age 20. About half (48.68%) of the respondents identified as women. Most of the respondents resided in urban areas (77.95%), were never married (79.87%), had high school and below degree (98.87%), and were employed (87.72%) at age 20. More than half of the respondents were insured (71.08%) and 91.24% reported excellent to good health status when aged 20. The mean household income was 73,100 in 2015 US dollars. Mean household size was about four and the mean parental education was 13.73 years.

**Table 1:** Demographic and socioeconomic characteristics of respondents when aged 20.

	Whole Sample (n = 8,984)	Non-Hispanic White (n = 4,406)	Non-Hispanic Black (n = 2,335)	Hispanic (n = 1,901)
*Categorical variable*	*%*			
Race/ethnicity				
Non-Hispanic White	66.54			
Non-Hispanic Black	15.41			
Hispanic	12.86			
Other or multiracial non-Hispanic†	5.20			
Health				
Excellent/very good/good health	91.24	92.20	88.88	89.17
Fair/poor health	8.76	7.80	11.12	10.83
Gender				
Male	51.32	51.07	50.98	53.61
Female	48.68	48.93	49.02	46.39
Region				
Northeast	16.98	18.58	13.59	14.55
North central	24.78	29.76	17.40	10.67
South	36.50	32.94	61.12	29.79
West	21.74	18.73	7.88	44.99
Geographic area				
Rural	22.05	27.29	15.68	7.75
Urban	77.95	72.71	84.32	92.25
Employment status				
No	12.28	10.67	19.28	11.91
Yes	87.72	89.33	80.72	88.09
Insurance status				
No	28.92	25.27	37.44	36.56
Yes	71.08	74.73	62.56	63.44
Education				
High School and below	98.87	98.74	99.32	98.82
Associate/junior/bachelor's degree	1.13	1.26	0.68	1.18
Marital status				
Never married, not cohabitating	79.87	78.80	86.91	74.23
Married/cohabitating	19.71	20.73	12.94	25.14
Separated/divorced/widowed, not cohabitating	0.42	0.47	0.15	0.64
*Continuous variable*	*Mean, (SD)*			
Income (10k)	7.31 (7.29)	8.00 (7.70)	5.03 (5.88)	6.63 (6.29)
Parental education	13.73 (2.89)	14.28 (2.59)	12.94 (2.15)	11.60 (3.68)
Household size	3.59 (1.63)	3.42 (1.48)	3.74 (1.81)	4.09 (1.87)

Note: Results are weighted; Race/ethnicity, gender, and parental education were measured at the baseline (1997), all other characteristics were measured when respondents were aged 20. †:Other or multiracial non-Hispanic group (unweighted N = 342) includes American Indian and Alaska Native (n = 43), Asian or Pacific Islander (n = 156), Mixed race (non-Hispanic) (n = 83), and something else (n = 60).

### 3.2 Racial wealth disparities

Table 2 presents wealth quartile cut points for each racial/ethnic group when respondents were aged 20, 25, and 30. Significant racial/ethnic wealth disparities across racial/ethnic groups were observed in young adulthood. For example, non-Hispanic White respondents’ median parental net worth, measured in 1997, was 5.82 times greater than that of Black households’ and 4.90 times greater than that of the Hispanic households. Non-Hispanic White respondents’ median self-reported net worth at age 20 was 2.76 times that of non-Hispanic Black households’ and 1.25 times that of the Hispanic households. For ages 25 and 30, this trend persisted, with White respondents’ net worth approximately 2.81 (2.35) times that of Black households and 1.27 (1.23) times greater than that of Hispanic households, respectively.

**Table 2:** Racial wealth disparities across young adulthood (wealth quartile cut points).

	Whole sample	Non-Hispanic White	Non-Hispanic Black	Hispanic
Parental net worth, 1997 (time-invariant)				
Net worth (10k), continuous measure	Median, Mean (SD)	Median, Mean (SD)	Median, Mean (SD)	Median, Mean (SD)
	7.655, 16.236 (20.210)	11.294, 19.797 (23.698)	1.941, 5.552 (10.912)	2.303, 8.114 (14.896)
Net worth (10k) Quartile	Cut points (%)	Cut points (%)	Cut points (%)	Cut points (%)
Q1 (lowest)	<=0.827 (19.59%)	<=3.126 (25.06%)	<=0.391 (24.67%)	<=0.332 (23.21%)
Q2	0.827-5.093 (22.94%)	3.126-11.191 (24.90%)	0.391-1.624 (23.63%)	0.332-1.698 (22.29%)
Q3	5.093-16.941 (26.42%)	11.191-27.150 (24.99%)	1.624-5.928 (25.51%)	1.698-8.046 (26.41%)
Q4 (highest)	>16.941 (31.05%)	>27.150 (25.05%)	>5.928 (26.18%)	>8.046 (28.09%)
Self-reported net worth, age 20				
	Median, Mean (SD)	Median, Mean (SD)	Median, Mean (SD)	Median, Mean (SD)
Net worth (10k), continuous measure	0.943, 3.482 (11.902)	1.105, 4.170 (9.807)	0.400,1.646 (5.206)	0.881, 2.914 (8.551)
Net worth (10k) Quartile	Cut points (%)	Cut points (%)	Cut points (%)	Cut points (%)
Q1 (lowest)	<=0.286 (24.31%)	<=.322 (24.98%)	<=0.250 (25.80%)	<=0.313 (25.72%)
Q2	0.286-0.843 (23.07%)	.322-1.102 (25.01%)	0.250-0.400 (24.31%)	0.313-0.857 (23.54%)
Q3	0.843-2.852 (25.03%)	1.102-4.222 (25.04%)	0.400-1.651 (25.22%)	0.857-2.409 (24.62%)
Q4 (highest)	>2.852 (27.59%)	>4.222(24.97%)	>1.651 (24.67%)	>2.409 (26.12%)
Self-reported net worth, age 25				
	Median, Mean (SD)	Median, Mean (SD)	Median, Mean (SD)	Median, Mean (SD)
Net worth (10k), continuous measure	0.941, 3.691 (9.698)	1.120, 4.252 (10.511)	0.399, 1.854 (6.176)	0.882, 3.048 (8.353)
Net worth (10k) Quartile	Cut points (%)	Cut points (%)	Cut points (%)	Cut points (%)
Q1 (lowest)	<=0.276 (24.41%)	<=.322 (25.67%)	<=0.250 (26.05%)	<=0.303 (24.95%)
Q2	0.276-0.828 (23.09%)	.322-1.120 (24.42%)	0.250-0.395 (23.87%)	0.303-0.863 (24.71%)
Q3	0.828-2.744 (24.99%)	1.120-3.951 (24.90%)	0.395-1.429 (24.68%)	0.863-2.574 (24.75%)
Q4 (highest)	>2.744 (27.50%)	>3.951 (25.01%)	>1.429 (25.40%)	>2.574 (25.59%)
Self-reported net worth, age 30				
	Median, Mean (SD)	Median, Mean (SD)	Median, Mean (SD)	Median, Mean (SD)
Net worth (10k), continuous measure	0.941, 3.693 (9.427)	1.067, 4.165 (9.994)	0.454, 1.994 (6.423)	0.864, 3.214 (9.053)
Net worth (10k) Quartile	Cut points (%)	Cut points (%)	Cut points (%)	Cut points (%)
Q1 (lowest)	<0.286 (24.12%)	<=.322 (25.25%)	<=0.254 (26.38%)	<=0.303 (24.69%)
Q2	0.286-0.847 (23.38%)	.322-1.070 (24.89%)	0.254-0.425 (22.73%)	0.303-0.856 (24.87%)
Q3	0.847-2.746 (25.14%)	1.070-4.100 (24.89%)	0.425-1.528 (24.98%)	0.856-2.391 (25.29%)
Q4 (highest)	>2.746 (27.36%)	>4.100 (24.96%)	>1.529 (25.91%)	>2.391 (25.14%)

Note: Results are weighted. Inflation was adjusted based on Consumer Price Index, in 2015 US dollars.

### 3.3 Whole sample multilevel analyses results

Table 3 presents mixed effects model results, which predicted SRH using the whole sample. Model 1 shows that, compared to non-Hispanic White, non-Hispanic Black (*OR* = 0.664, 95% *CI* = 0.540, 0.817), Hispanic (*OR* = 0.741, 95% *CI* = 0.586, 0.938), and other or multiracial non-Hispanic (*OR* = 0.651*, 95% CI =* 0.427, 0.994) had significantly lower odds of reporting excellent/very good/good health. Income (*OR* = 1.020*, 95% CI =* 1.006, 1.034) and employment (*OR* = 1.967*, 95% CI* = 1.626, 2.379 significantly predicted SRH. Each additional year of parental education was associated with 7.5% higher odds of respondents reporting excellent/very good/good health (*OR* = 1.075, 95% *CI* = 1.041, 1.110). Regarding education, compared to high school and below, associate/junior/bachelor’s degree (*OR* = 2.457, 95% *CI* = 1.878, 3.214) and master’s/PhD/professional degree (*OR* = 4.708, 95% *CI* = 2.178, 10.180) strongly predicted SRH. In addition, insurance (*OR* = 1.314, 95% *CI* = 1.124, 1.535) significantly predicted SRH at 0.05 level according to Model 1.

**Table 3:** Two-level logistic model for excellent/very good/good health, whole sample.

	Model 1	Model 2
	Odds Ratio (95% CI)	Odds Ratio (95% CI)
**Fixed effects**		
**Household socioeconomic position indicators**		
Net worth (Ref: Q1, lowest)		
Q2		**1.904 (1.541, 2.352)**
Q3		**1.833 (1.475, 2.277)**
Q4 (highest)		**2.297 (1.806, 2.921)**
Parental net worth in 1997 (Ref: Q1, lowest)		
Q2		**1.462 (1.142, 1.871)**
Q3		**1.611 (1.233, 2.105)**
Q4 (highest)		**2.779 (2.018, 3.827)**
Race/ethnicity(Ref: Non-Hispanic White)		
Non-Hispanic Black	**0.664 (0.540, 0.817)**	0.817 (0.649, 1.029)
Hispanic	**0.741 (0.586, 0.938)**	0.782 (0.610, 1.003)
Other or multiracial non-Hispanic	**0.651 (0.427, 0.994)**	0.756 (0.473, 1.207)
Income (10k USD, 2015)	**1.020 (1.006, 1.034)**	1.008 (0.993, 1.023)
Employment (Ref: No)		
Yes	**1.967 (1.626, 2.379)**	**1.871 (1.523, 2.300)**
Education (Ref: High School)		
Associate/junior/bachelor's degree	**2.457 (1.878, 3.214)**	**2.197 (1.641, 2.939)**
Master/PhD/Professional degree	**4.708 (2.178, 10.180)**	**3.306 (1.481, 7.380)**
Parental education	**1.075 (1.041, 1.110)**	1.033 (0.997, 1.071)
**Household demographic characteristics**		
Region (Ref: Northwest)		
North central	0.907 (0.704, 1.169)	0.933 (0.708, 1.229)
South	1.171 (0.928, 1.478)	1.210 (0.941, 1.555)
West	1.157 (0.890, 1.504)	1.208 (0.909, 1.472)
Geographic area (Ref: Rural)		
Urban	1.020 (0.848, 1.227)	1.031 (0.846, 1.296)
Insurance (Ref: not insured)		
Insured	**1.314 (1.124, 1.535)**	1.163 (0.981, 1.378)
Wave (Ref: Aged 20)		
Aged 25	0.991 (0.711, 1.169)	1.084 (0.786, 1.494)
Aged 30	1.033 (0.468, 2.279)	1.105 (0.535, 2.280)
Household size	1.014 (0.969, 1.062)	1.026 (0.977, 1.078)
Gender (Ref: Male)		
Female	**0.683 (0.579, 0.807)**	**0.740 (0.620, 0.884)**
Marital status (Ref: Never married)		
Married/cohabitating	1.066 (0.901, 1.261）	0.974 (0.810, 1.171)
Separated/divorced/widowed, not cohabitating	1.025 (0.688, 1.528)	0.901 (0.585, 1.388)
Constant	**8.894 (3.105, 11.186)**	**3.758 (1.932, 7.312)**
**Random effects**		
Variance of intercept	**3.251 (2.055, 5.143)**	**3.112 (2.016, 4.805)**
Variance of wave slope	0.106 (0.003, 4.345)	0.113 (0.004, 2.849)
Number of observations	22,410	18,858
Number of respondents	8,512	8,270

Note: Results are weighted and based on 20 imputed datasets after applying the Rubin’s rule. Bold indicates statistical significance at 0.05 level.

Model 2 added net worth quartiles measured from the respondents and their parental net worth. Overall, findings indicated a strong, positive association between wealth and SRH, with each quartile significantly associated with SRH compared to the lowest quartile. For example, respondents in the highest net worth quartile had more than 2 times greater odds of having excellent/very good/good health (*OR* = 2.297*, 95% CI =* 1.806, 2.921), relative to respondents in the lowest wealth quartile. Each parental net worth quartile was also significantly associated with SRH, with the highest quartile almost 3 times more likely to report excellent/very good/good health compared to the lowest quartile (*OR* = 2.779*, 95% CI =* 2.018, 3.827).

After controlling for respondents’ self-reported net worth and parental net worth, racial/ethnic differences in SRH were no longer statistically significant. Observed gender differences in SRH were also reduced after accounting for parental and individuals’ net worth. Wealth also reduced SEP disparities in health measured by education and employment. Notably, income, parental education, and insurance status were no longer significantly associated with SRH after controlling for respondents’ net worth and parental net worth.

### 3.4 Racial/ethnic subgroup multilevel analyses results

Table 4 presents racial/ethnic subgroup analyses results. Overall, findings indicated positive associations between wealth and SRH across racial/ethnic groups. However, there were notable variations in the patterning of the association between racial/ethnic groups.

**Table 4:** Two-level logistic model for excellent/very good/good health, racial/ethnic differences.

	Non-Hispanic White	Non-Hispanic Black	Hispanic
	Odds Ratio (95% CI)	Odds Ratio (95% CI)	Odds Ratio (95% CI)
**Fixed effects**			
**Household socioeconomic position indicators**			
Net worth (Ref: Q1, lowest)			
Q2	**1.841 (1.409, 2.405)**	1.313 (0.963, 1.792)	**2.631 (1.840, 3.764)**
Q3	**1.952 (1.439, 2.648)**	1.214 (0.887, 1.661)	**2.120 (1.470, 3.056)**
Q4 (highest)	**2.335 (1.650, 3.305)**	**1.727 (1.198, 2.491)**	**1.960 (1.296, 2.964)**
Parental net worth in 1997 (Ref: Q1, lowest)			
Q2	1.219 (0.868, 1.713)	0.989 (0.691, 1.417)	1.110 (0.708, 1.740)
Q3	**1.881 (1.276, 2.773)**	1.225 (0.820, 1.830)	0.970 (0.616, 1.527)
Q4 (highest)	**2.133 (1.326, 3.430)**	**1.749 (1.095, 2.794)**	1.541 (0.915, 2.595)
Income (10k USD, 2015)	1.011 (0.993, 1.030)	1.007 (0.981, 1.033)	**1.028 (1.000, 1.056)**
Employment (Ref: No)			
Yes	**2.456 (1.827, 3.302)**	**1.536 (1.162, 2.029)**	**1.484 (1.026, 2.145)**
Education (Ref: High School)			
Associate/junior/bachelor's degree	**2.785 (1.898, 4.085)**	1.288 (0.873, 1.900)	**2.079 (1.252, 3.451)**
Master/PhD/Professional degree	**4.798 (1.645, 13.992)**	**7.075 (1.839, 27.216)**	3.779 (0.681, 20.978)
Parental education	**1.074 (1.015, 1.137)**	**1.073 (1.003, 1.148)**	0.993 (0.950, 1.038)
**Household demographic characteristics**			
Region (Ref: Northwest)			
North central	0.860 (0.609, 1.214)	0.832 (0.539, 1.282)	1.193 (0.625, 2.279)
South	1.011 (0.721, 1.418)	**1.601 (1.106, 2.316)**	1.625 (0.997, 2.647)
West	1.116 (0.753, 1.654)	1.433 (0.791, 2.597)	1.089 (0.689, 1.722)
Geographic area (Ref: Rural)			
Urban	1.006 (0.790, 1.282)	1.042 (0.741, 1.466)	1.432 (0.895, 2.292)
Insurance (Ref: not insured)			
Insured	**1.388 (1.091, 1.767)**	1.013 (0.787, 1.304)	0.986 (0.738, 1.318)
Wave (Ref: Aged 20)			
Aged 25	1.188 (0.730, 1.934)	0.961 (0.742, 1.244)	1.003 (0.740, 1.358)
Aged 30	1.553 (0.526, 4.581)	0.861 (0.653, 1.135)	1.151 (0.817, 1.623)
Household size	1.004 (0.928, 1.086)	1.036 (0.971, 1.106)	1.016 (0.945, 1.092)
Gender (Ref: Male)			
Female	**0.744 (0.579, 0.957)**	**0.581 (0.439, 0.769**)	0.908 (0.670, 1.230)
Marital status (Ref: Never married)			
Married/cohabitating	1.108 (0.860, 1.429)	1.001 (0.767, 1.306)	0.952 (0.670, 1.230)
Separated/divorced/widowed, not cohabitating	1.508 (0.786, 2.894)	1.573 (0.650, 3.808)	**0.414 (0.218, 0.785)**
Constant	2.305 (0.862, 6.162)	**2.943 (1.094, 7.914)**	**3.757 (1.449, 9.741)**
**Random effects**			
Variance of intercept	**3.075 (1.646, 5.743)**	**2.943 (1.094, 7.914)**	**2.629 (1.868, 3.700)**
Variance of wave slope	0.324 (0.059, 1.796)	**0.000 (0.000, 0.000)**	**0.000 (0.000, 0.000)**
Number of observations	10,937	6,008	4,679
Number of respondents	4,151	2,237	1,808

Note: Results are weighted and based on 20 imputed datasets after applying the Rubin’s rule. Bold indicates statistical significance at 0.05 level.

#### 3.4.1 Racial/ethnic differences in young adulthood time-varying wealth and health

For non-Hispanic White and Hispanic respondents, we observed incremental improvements in SRH as net worth quartile increased. Compared to the lowest net worth quartile, the second quartile (*OR* = 1.814*, 95% CI =* 1.409, 2.405), third quartile (*OR* = 1.952*, 95% CI =* 1.439, 2.648), and highest quartile (*OR* = 2.335*, 95% CI =* 1.650, 3.305) were all associated with higher odds of better health among non-Hispanic White respondents. Similarly, among Hispanic respondents, young adults in the second quartile (*OR* = 2.631, 95% *CI =* 1.840, 3.764), third quartile (*OR* = 2.120, 95% *CI =* 1.470, 3.056), and highest quartile (*OR* = 1.960, 95% *CI =* 1.296, 2.964) all had greater odds of better SRH, after controlling for other variables in the model. However, among non-Hispanic Black respondents, only respondents in the highest wealth quartile (*OR* = 1.727*, 95% CI =* 1.198, 2.491) had significant association of better SRH, compared to the lowest net worth quartile and other variables being held constant.

#### 3.4.2 Racial/ethnic differences in parental wealth and health

Notable differences appeared between the associations between parental net worth and SRH across racial/ethnic groups. For non-Hispanic Black respondents, parental net worth in 1997 (baseline) was only significantly associated with SRH among the highest quartile (*OR* = 1.749*, 95% CI =* 1.095, 2.794). By contrast, parental net worth did not significantly predict SRH in any of the wealth quartiles among Hispanic respondents. For non-Hispanic White respondents, however, parental net worth significantly predicted SRH among the highest (*OR* = 2.133*, 95% CI =* 1.326, 3.430) and second to the highest quartile (*OR* = 1.881*, 95% CI* = 1.276, 2.773).

Respondents’ household income was not significantly associated with SRH among non-Hispanic White and non-Hispanic Black samples given net worth measures were in the model; whereas employment status significantly predicts SRH across all racial/ethnic groups at the 0.05 level (*OR* non-Hispanic White = 2.456; *OR* non-Hispanic Black = 1.536; *OR* Hispanic = 1.484). Moreover, parental education only significantly predicts respondents’ SRH among the non-Hispanic While sample (*OR* = 1.074*, 95% CI =* 1.015, 1.137). Similarly, insurance status was significantly associated with SRH among the non-Hispanic White sample (*OR* = 1.388*, 95% CI* = 1.091, 1.767) but not among non-Hispanic Black sample and Hispanic.

The association between education and SRH varied across racial/ethnic groups. Positive associations were found among non-Hispanic White sample, with both associate/junior/bachelor's degrees (*OR* = 2.785*, 95% CI =* 1.898, 4.085) and graduate/professional degrees (*OR* = 4.798*, 95% CI =* 1.645, 13.992) associated with better SRH, compared to respondents’ education levels of high school and below. However, different patterning was found in the other two groups. For example, when compared to high school and below education level, non-Hispanic Black respondents who had graduate/professional degrees had 7 times higher odds of having better health (*OR* = 7.075*, 95% CI =* 1.839, 27.216). Furthermore, those with associate/junior/bachelor's degrees did not have significantly different SRH among the non-Hispanic Black sample, compared to those who completed high school or below. For the Hispanic sample, however, graduate/professional degrees did not significantly predict SRH, whereas associate/junior/bachelor's degree was associated with better health (*OR* = 2.079*, 95% CI =* 1.252, 3.451).

Although marital status was not associated with SRH in the whole sample or among the non-Hispanic White and Black sample, significant associations were found between separated/divorced/widowed who are not cohabitating and SRH among the Hispanic sample. Compared to the never married group, those who were separated/divorced/widowed and not cohabitating were less likely to report better health (*OR* = 0.414*, 95% CI =* 0.218, 0.785). Moreover, significant gender differences were observed among non-Hispanic White (*OR* female = 0.744) and non-Hispanic Black (*OR* female = 0. 581), but not within the Hispanic sample.

## 4 Discussion

This study examined racial/ethnic differences in longitudinal relationship between wealth and health during young adulthood, with commonly studied SEP indicators (income, education, employment) being controlled for. We found notable racial/ethnic differences in the patterning of wealth–health associations that appear to emerge in early adulthood. Our findings also indicate that there are significant racial differences in the relationship between parental wealth and health. The findings have several implications for research, policy, and practice.

First, our findings indicate that wealth is an important SEP indicator as results show that the effects of wealth on health are different from the effects of income, education, and employment. In our whole sample analysis, after controlling for parental wealth and young adults’ individual wealth, racial differences in SRH were no longer statistically significant. During young adulthood, wealth appeared to be a stronger factor related to health than other SEP factors, pointing to the importance of wealth in research and intervention. Further, after wealth was added to the model, household income, parental education, and insurance status were no longer significantly associated with SRH.

Second, these findings suggest that race/ethnicity is inextricably linked to wealth and health inequities. We observed different patterns of the relationship between wealth and health across racial/ethnic groups. For self-reported net worth during young adulthood, incremental increases in the wealth quartile were associated with better SRH among non-Hispanic White respondents and Hispanics, but not among Black respondents. In contrast, for parental net worth and individuals’ own net worth, wealth was only significantly associated with health among the highest wealth quartiles for non-Hispanic Black respondents. Furthermore, parental net worth was not associated with health in any parental wealth quartile among the Hispanic sample.

Our findings observed nuanced relationships between wealth and health within racial/ethnic groups that otherwise cannot be captured by only using whole sample analysis with race/ethnicity controlled for. Due to historical racial wealth inequities, the findings from this study emphasize the importance of examining the effects of wealth on health by intentionally accounting for the effects of racism (Hudson, 2021). Race is primary in the conceptualization of social determinants of health (Sun et al., 2021). Researchers that investigate socioeconomic and health inequities should consider not simply treat race/ethnicity as surveillance or control variables. Instead, racism should be explicitly conceptualized and measured as fundamental determinants of health inequities (Nuru-Jeter et al., 2018; Syme, 2008; Williams, Lawrence, & Davis, 2019). Recent advancement in health equity research calls for measuring racism at the structural level (e.g., Adkins-Jackson et al., 2022; Hardeman et al., 2022). Indeed, redressing historical legacies of racial violence requires a critical, race conscious lens and uplifting experiences of the most marginalized (Hudson, 2021).

Third, greater variations were found across racial/ethnic groups in the associations between intergenerational wealth and health compared to time-varying self-reported wealth in young adulthood. It is not surprising that the wealth gap across racial/ethnic groups is more prominent in parental net worth: non-Hispanic White respondents’ median parental net worth measured in 1997 was almost six times that of Black households and five times that of the Hispanic households. By contrast, the observed racial wealth gaps from ages 20–30 were much smaller. At the current rate of growth, the wealth gap between White American families and Black and Latino families will have doubled, on average, from about $500,000 in 2013 to over $1 million by 2044 (Asante-Muhammed et al., 2016), when people of color are predicted to compose majority of the U.S. population (Frey, 2014)

Fourth, this study found that associations between other SEP indicators and SRH vary by race/ethnicity. Having an associate’s or bachelor’s degree was significantly associated with greater SRH among non-Hispanic White and Hispanic respondents but not among Black respondents. Prior research, however, indicates that even with the same level of educational attainment, there are substantial differences that researchers should consider such as the perceived prestige of academic institutions that were attended as well as social capital inhered within social networks. These factors can affect SEP (Williams & Collins, 2001). Similarly, parental education was only significantly associated with health among non-Hispanic White respondents, outlining the effects of intergenerational SEP advantage on health among White Americans.

Fifth, this study suggested some socioeconomic factors that should further be investigated among Hispanic Americans. We found that respondents’ self-reported net worth, not parental net worth, was associated with SRH. Similarly, education level did not significantly predict health among Hispanics. In addition, gender was not significantly associated with SRH, whereas marital status had a significant and a large effect on SRH among Hispanic respondents.

There are several potential explanations for these findings related to Hispanics. First, SRH among the Hispanic populations may vary by several factors that were not available in these data such as immigration status, generational status, length of stay, language, and other factors (Finch et al., 2002; Kandula, Lauderdale, & Baker, 2007). Second, previous research has indicated that cultural and contextual factors may be protective of low SEP within Hispanic communities, including the presence of an ethnic enclave residence (Logan, Zhang, & Alba, 2002.; Melvin et al., 2014; Viruell-Fuentes et al., 2013); nativity status differences across first, second, and third generation Hispanic immigrants (Melvin et al., 2014; Viruell-Fuentes, 2011); and country of origin and cultural factors (Abraído-Lanza et al., 1999; Franzini & Keddie, 2001; Markides & Eschbach, 2011).

## 5 Limitations

This study has several limitations. First, small sample sizes preclude the subgroup examination of other racial/ethnic groups, such as Native Americans and Asian Americans and Pacific Islander. For example, previous research indicates that wealth inequality and health disparities persist among Asian Americans (Gee & Ford, 2011; Weller & Thompson, 2018). Neither could we decompose the Hispanic sample into subgroups based on nativity or nationality. Future studies should oversample these populations to investigate the racial/ethnic differences between wealth as health. Second, this study operationalized wealth as net worth. Nonetheless, there are subcomponents of wealth, such as various types of assets and debt, that could affect health in different ways. Future research should dissect wealth components and study their links to health outcomes to inform effective ways to build assets and relieve debt. Additionally, wealth is difficult to measure as respondents may have difficulty estimating wealth or debt, especially on surveys without access to their financial records. It is also difficult to collect data from very high earning, high wealth individuals, therefore the wealth-health association may be even more significant than currently estimated. Third, we operationalized health as SRH. Although research has demonstrated SRH is predictive of all-cause mortality and other health outcomes, other health outcomes may have different relationships with SRH. Future studies should collect both wealth and more health outcome measures to enable studies on the relationship between wealth and health. Fourth, as indicated above, SRH may vary across racial/ethnic groups, which may affect whole sample results but not within racial/ethnic group comparisons. However, research also shows lower reliability of SRH among low education and income groups (Zajacova & Dowd, 2011). Furthermore, there is time-varying confounding in this study. For example, income can serve as both a confounder at one time and a mediator at a later time point. Future research should consider using marginal structural models or other causal inference models to account for this. Finally, we failed to account for other confounding factors that were not measured in this study, such as neighborhood SES, education quality, and occupation prestige.

## 6 Conclusion

Notwithstanding these limitations, this study extends the current knowledge by examining the longitudinal SEP-health gradient during young adulthood between and within racial/ethnic groups in the US. As demonstrated in this study and others, there are substantial intergenerational racial/ethnic wealth gaps. These gaps may be remedied by the adoption of inclusive, asset-building policies. Most U.S. asset-based policies (e.g., home mortgage interest deduction and retirement accounts) deliver through tax benefits and disproportionately benefit middle- and high-income earners (Greer & Levin, 2014). Such mechanisms pose institutional barriers to disadvantaged families and people of color who do not own a home nor have employment benefits such as retirement accounts. Ongoing policy innovations call for inclusive and universal asset building vehicles to address these barriers and inequities. For example, progressive features in Child Development Account (CDA) policies (Sherraden et al., 2019) include larger initial deposits and/or additional deposits for the poorest children over time, as well as greater savings matches for financially vulnerable families. Findings also suggest policy proposals such as “baby bonds” (Darity & Hamilton, 2012) and reparations (Bassett & Galea, 2020; Williams & Collins, 2004) as ways to eliminate racial wealth gap to narrow racial health inequities. Further, these findings suggest the importance of measuring wealth in health studies to inform practice and policy. Policies that provide progressive monetary subsidies to compensate for disadvantage may narrow the socioeconomic and health inequalities in the U.S.

## Funding

This research receives partial funding support from the Jane B. Aron Doctoral Fellowship from The National Association of Social Workers Foundation.

## Availability of data and material

Data used in this research can be downloaded at: https://www.nlsinfo.org/content/cohorts/nlsy97.

## Consent for publication

The author has approved the manuscript and agree with its submission to the journal.

## Disclosure of potential conflicts of interest

The author is not aware of any affiliations, memberships, funding, or financial holdings that might be perceived as affecting the objectivity of this research.

## Research involving human participants and/or animals

Not applicable.

## Informed consent

Not applicable. This research uses secondary data.

## Credit author statement

Sicong Sun: Conceptualization, Methodology, Formal analysis, Writing - original draft, Writing - review & editing, Funding acquisition.

Hedwig Lee: Writing - review & editing.

Darrell Hudson: Conceptualization, Writing - review & editing.

## Data Availability

The data is publicly available
