# Supplementary material for: Racial/ethnic differences in the relationship between wealth and health across young adulthood
Source: SSM Popul Health. 2022 Dec 20;21:101313. doi: 10.1016/j.ssmph.2022.101313 (PMC9798167; doi:10.1016/j.ssmph.2022.101313)
Supplement: Multimedia component 1 [file mmc1.docx]

Table S1. Demographic and Socioeconomic Characteristics of Respondents When Aged 25

|  | Whole Sample (n=8,984) | Non-Hispanic White (n=4,406) | Non-Hispanic Black (n=2,335) | Hispanic (n=1,901) |
| --- | --- | --- | --- | --- |
| *Categorical variable* | *%* |  |  |  |
| Race/ethnicity |  |  |  |  |
| Non-Hispanic White | 66.54 |  |  |  |
| Non-Hispanic Black | 15.41 |  |  |  |
| Hispanic | 12.86 |  |  |  |
| Other or multiracial non-Hispanic | 5.20 |  |  |  |
| Health |  |  |  |  |
| Excellent/very good/good health | 91.83 | 93.15 | 88.82 | 89.12 |
| Fair/poor health | 8.17 | 6.85 | 11.18 | 10.88 |
| Gender |  |  |  |  |
| Male | 51.32 | 51.07 | 50.98 | 53.61 |
| Female | 48.68 | 48.93 | 49.02 | 46.39 |
| Region |  |  |  |  |
| Northeast | 16.66 | 18.13 | 13.43 | 14.59 |
| North central | 24.83 | 29.58 | 17.79 | 11.54 |
| South | 37.13 | 32.62 | 61.50 | 31.00 |
| West | 21.39 | 18.67 | 7.27 | 42.86 |
| Geographic area |  |  |  |  |
| Rural | 21.83 | 26.91 | 14.58 | 9.54 |
| Urban | 78.17 | 73.09 | 85.42 | 90.46 |
| Employment status |  |  |  |  |
| No | 12.41 | 10.44 | 18.79 | 13.12 |
| Yes | 87.59 | 89.56 | 81.21 | 86.88 |
| Insurance status |  |  |  |  |
| No | 29.10 | 25.45 | 37.80 | 37.03 |
| Yes | 70.90 | 74.55 | 62.20 | 62.97 |
| Education |  |  |  |  |
| High School and below | 68.19 | 63.18 | 81.97 | 82.24 |
| Associate/junior/bachelor's degree | 29.83 | 34.34 | 16.88 | 17.20 |
| Master/PhD/professional degree | 1.98 | 2.48 | 1.15 | 0.56 |
| Marital status |  |  |  |  |
| Never married, not cohabitating | 48.93 | 44.03 | 66.90 | 46.30 |
| Married/cohabitating | 47.77 | 52.53 | 30.70 | 49.78 |
| Separated/divorced/widowed, not cohabitating | 3.30 | 3.44 | 2.40 | 3.92 |
| *Continuous variable* | *Mean, (SD)* |  |  |  |
| Income (10k) | 7.40 (7.32) | 8.04 (7.65) | 5.05 (5.95) | 6.53 (5.57) |
| Parental education | 13.73 (2.89) | 14.28 (2.59) | 12.94 (2.15) | 11.60 (3.68) |
| Household size | 3.00 (1.54) | 2.82 (1.38) | 3.20 (1.69) | 3.63 (1.85) |

Note: Results are weighted; Race/ethnicity, gender, and parental education were measured at the baseline (1997), all other characteristics were measured when respondents were aged 25.

Table S2. Demographic and Socioeconomic Characteristics of Respondents When Aged 30

|  | Whole Sample (n=8,984) | Non-Hispanic White (n=4,406) | Non-Hispanic Black (n=2,335) | Hispanic (n=1,901) |
| --- | --- | --- | --- | --- |
| *Categorical variable* | *%* |  |  |  |
| Race/ethnicity |  |  |  |  |
| Non-Hispanic White | 66.54 |  |  |  |
| Non-Hispanic Black | 15.41 |  |  |  |
| Hispanic | 12.86 |  |  |  |
| Other or multiracial non-Hispanic | 5.20 |  |  |  |
| Health |  |  |  |  |
| Excellent/very good/good health | 91.85 | 92.95 | 88.46 | 90.95 |
| Fair/poor health | 8.15 | 7.05 | 11.54 | 9.05 |
| Gender |  |  |  |  |
| Male | 51.32 | 51.07 | 50.98 | 53.61 |
| Female | 48.68 | 48.93 | 49.02 | 46.39 |
| Region |  |  |  |  |
| Northeast | 16.54 | 18.01 | 12.88 | 14.48 |
| North central | 24.64 | 29.76 | 16.71 | 10.94 |
| South | 37.26 | 33.67 | 62.59 | 30.55 |
| West | 21.55 | 18.56 | 7.82 | 44.03 |
| Geographic area |  |  |  |  |
| Rural | 21.76 | 26.60 | 16.14 | 8.34 |
| Urban | 78.24 | 73.40 | 83.86 | 91.66 |
| Employment status |  |  |  |  |
| No | 12.06 | 10.46 | 18.88 | 11.58 |
| Yes | 87.94 | 89.54 | 81.12 | 88.42 |
| Insurance status |  |  |  |  |
| No | 29.40 | 26.37 | 36.71 | 36.16 |
| Yes | 70.60 | 73.63 | 63.29 | 63.84 |
| Education |  |  |  |  |
| High school and below | 60.93 | 55.89 | 74.61 | 74.65 |
| Associate/junior/bachelor's degree | 30.54 | 34.00 | 20.49 | 21.19 |
| Master/PhD/professional degree | 8.53 | 10.11 | 4.90 | 4.16 |
| Marital status |  |  |  |  |
| Never married, not cohabitating | 31.67 | 25.26 | 52.97 | 31.59 |
| Married/cohabitating | 62.26 | 68.69 | 41.35 | 61.41 |
| Separated/divorced/widowed, not cohabitating | 6.07 | 6.05 | 5.68 | 7.01 |
| *Continuous variable* | *Mean, (SD)* |  |  |  |
| Income (10k) | 7.35 (7.31) | 8.04 (7.69) | 4.80 (5.28) | 6.61 (6.06) |
| Parental education | 13.73 (2.89) | 14.28 (2.59) | 12.94 (2.15) | 11.60 (3.68) |
| Household size | 3.19 (1.57) | 3.10 (1.46) | 3.2 (1.73) | 3.65 (1.78) |

Note: Results are weighted; Race/ethnicity, gender, and parental education were measured at the baseline (1997), all other characteristics were measured when respondents were aged 30.
